# Macrophages promote benzopyrene-induced tumor transformation of human bronchial epithelial cells by activation of NF-κB and STAT3 signaling in a bionic airway chip culture and in animal models

**DOI:** 10.18632/oncotarget.3561

**Published:** 2015-03-12

**Authors:** Encheng Li, Zhiyun Xu, Hui Zhao, Zhao Sun, Lei Wang, Zhe Guo, Yang Zhao, Zhancheng Gao, Qi Wang

**Affiliations:** ^1^ Department of Respiratory Medicine, The Second Affiliated Hospital, Dalian Medical University, Dalian, China; ^2^ Department of Physical Examination Center, The Second Affiliated Hospital, Dalian Medical University, Dalian, China; ^3^ The Liaoning Provincial Key Laboratory for Micro/Nano Technology, Dalian University of Technology, Dalian, China; ^4^ Department of Respiratory & Critical Care Medicine, The People's Hospital of Peking University, Beijing, China

**Keywords:** Macrophages, malignant transformation, NF-κB, STAT3, microfluidic chip

## Abstract

We investigated the role of macrophages in promoting benzopyrene (BaP)-induced malignant transformation of human bronchial epithelial cells using a BaP-induced tumor transformation model with a bionic airway chip *in vitro* and in animal models. The bionic airway chip culture data showed that macrophages promoted BaP-induced malignant transformation of human bronchial epithelial cells, which was mediated by nuclear factor (NF)-κB and STAT3 pathways to induce cell proliferation, colony formation in chip culture, and tumorigenicity in nude mice. Blockage of interleukin (IL)-6 or tumor necrosis factor (TNF)-α signaling or inhibition of NF-κB, STAT3, or cyclinD1 expression abrogated the effect of macrophages on malignant transformation in the bionic airway chip culture. *In vivo*, macrophages promoted lung tumorigenesis in a carcinogen-induced animal model. Similarly, blockage of NF-κB, STAT3, or cyclinD1 using siRNA transfection decreased the carcinogen-induced tumorigenesis in rats. We demonstrated that macrophages are critical in promoting lung tumorigenesis and that the macrophage-initiated TNF-α/NF-κB/cyclinD1 and IL-6/STAT3/cyclinD1 pathways are primarily responsible for promoting lung tumorigenesis.

## INTRODUCTION

Lung cancer is one of the leading causes of morbidity and mortality in the world, accounting for more than 1.6 million new cases and 1.3 million deaths each year [1]. Elucidation of the molecular mechanisms involved in lung tumorigenesis will lead to the emergence of novel diagnostic and therapeutic approaches against lung cancer. Accumulated knowledge shows that lung tumorigenesis is a complex process that includes multiple factors such as gene mutations or epigenetic changes, leading to uncontrolled cell proliferation, malignant transformation, and tumor metastasis. Other studies have demonstrated that chronic inflammation is closely associated with tumorigenesis in the liver and intestine [2, 3], while numerous epidemiological studies have indicated that chronic inflammation may also play a critical role in lung tumorigenesis [4]. However, the key molecules and signaling pathways that are involved in inflammation-mediated lung tumorigenesis remain to be fully elucidated.

Indeed, the chronic inflammatory microenvironment containing inflammatory cells, cytokines, and chemokines can promote malignant transformation of bronchial epithelial cells [5]. The persistent futile cycles of tissue injury and repair during chronic inflammation are a critical driver for lung cancer development and progression [6, 7]. Among these inflammatory cells in the lung, macrophages are the most abundant and the key components that link inflammation and cancer [8]. Infiltration of tumor-associated macrophages in tumor lesions is commonly found in breast cancer, hepatocellular carcinoma, colorectal cancer, and lung cancer and is associated with tumor angiogenesis, invasion, and metastasis [9-12]. To date, most studies have focused mainly on the role of macrophages in tumor progression based on an established tumor model with or without metastasis, but macrophages are rarely involved in tumor initiation [13-15].

Moreover, to further study the functions of macrophages in chronic airway inflammation-induced tumorigenesis, it is necessary to establish a biomimetic model that mimics the lung and airway microenvironment. Currently, the best model that is used frequently is 2D cell culture, which is far from the real environment *in vivo*. In addition, the microfluidic chip shows a great potential in biomimetic research and could be the ideal platform for studying tumorigenesis of the airway epithelium accompanied by macrophages [16, 17]. The microfluidic chip, a technology that features fluidic manipulation at the micrometer scale, has greatly improved biological and medical research [18]. Compared to traditional methods, microfluidics has numerous advantages like high throughput, integration, low cost, continuous flow, and real-time data analysis [19]. Recently, the microfluidic chip has developed towards an “organ-on-chip” model. The chips are commonly fabricated with versatile and controllable channels, various microvalves and micropumps, bioactive membranes, and integrated electrical or magnetic biosensors. In these organ-on-chip models, the key functions of an organ or the *in vivo* microenvironments are simulated by microfluidic engineering [17, 20, 21]. Hu *et al*. have successfully fabricated a lung chip to form an alveolar-capillary barrier to realize the exchange of gas and blood, thus providing a comprehensive tool for lung disease research [17].

In this study, we intended to construct a microfabricated airway mimic model to investigate the role of macrophages and the underlying mechanisms in chronic inflammation-mediated lung tumorigenesis. We first employed 57 human lung cancer tissues originating from the airways to assess the number of macrophages in lung cancer and the adjacent normal tissues. Next, we explored the potential tumorigenic role of macrophages and the underlying signaling pathways in a benzopyrene (BaP)-induced malignant transformation model with a fabricated bionic airway chip. After that, we performed an animal study to further confirm our *in vitro* data.

## RESULTS

### Distribution of macrophages in cancerous and adjacent lung tissues

The level of macrophage infiltration in lung tissue was assessed by immunostaining of CD68 protein in 57 formalin-fixed paraffin-embedded lung specimens. The number of CD68-positive cells in the lung cancer tissues was 119.56 ± 5.35, which was significantly higher than that in the adjacent normal lung cancer tissues (26.75 ± 10.38; n = 57, *p* < 0.05; Fig. 2). Furthermore, the number of CD68-positive cells in the lung cancer tissues was closely associated with the pathological type and tumor cell differentiation (*p* < 0.05; Table 1).

**Table 1:** Association of CD68-positive cells with clinicopathological factors of lung cancer patients (n = 57)

Clinicopathological characteristics	N	CD68^+^ cell number	*p* value
**Age (yrs.)**			
≥ 60	38	118.68 ± 5.12	0.298
< 60	19	120.46 ± 6.25	
**Sex**			
Male	40	118.33 ± 3.58	0.352
Female	17	119.77 ± 4.15	
**Pathological type**			
Squamous cell carcinoma	48	116.52 ± 7.15	0.001
Adenocarcinoma	9	122.35 ± 2.43	
**Tumor differentiation**			
Well-Moderate	42	117.15 ± 5.09	0.01
Poor	15	121.26 ± 6.11	
**TNM stage**			
I + II	50	118.07 ± 4.19	0.142
III + IV	7	120.16 ± 5.33	

**Figure 1:**
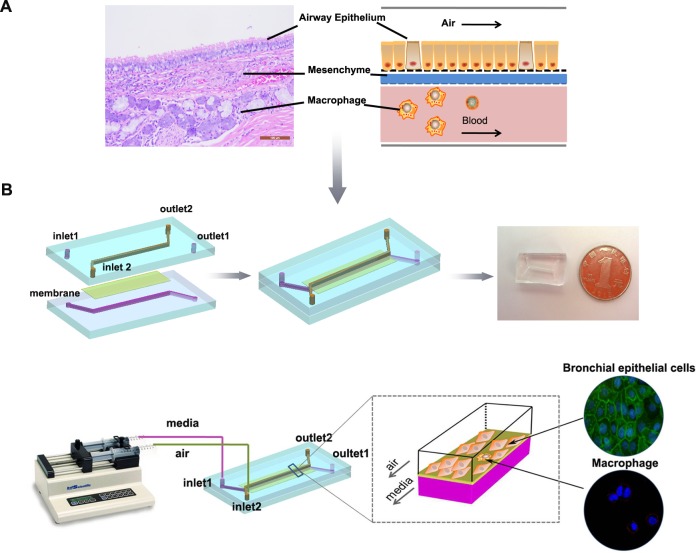
Illustration of the bionic airway chip A, Illustration of the airway structure and biomimetic model, including three vertical compartments with two different types of cells separated by porous membranes. B, Configuration of the bionic airway chip. The main body of the chip contains two polydimethylsiloxane layers and a piece of porous membrane. Sterile air and culture media flow in the top and bottom channels, respectively. The bronchial epithelial cells were grown on the top of the membrane, while the macrophages were grown on the opposite site of the porous membrane.

**Figure 2:**
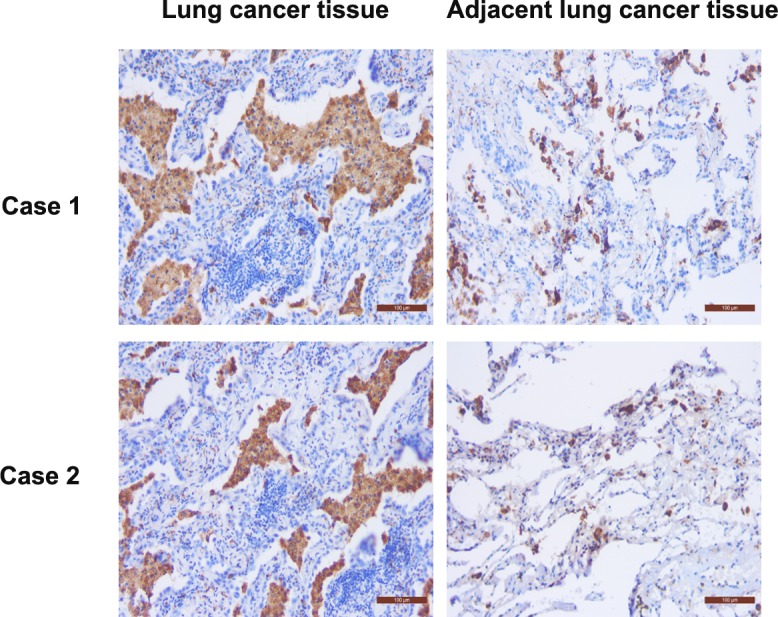
Immunostaining of CD68 protein in the cancerous and adjacent lung tissues from two lung cancer patients CD68 was mainly detected in the cytoplasm of macrophages, as shown by the brown color. The number of CD68-positive cells in the lung cancer tissue was more than that in the adjacent tissues (n = 57; *p* < 0.05; scale bar, 100 μm).

### Macrophage promotion of malignant transformation of human bronchial epithelial cells mediated by the NF-κB and STAT3 pathways in the bionic airway chip

**Figure 3:**
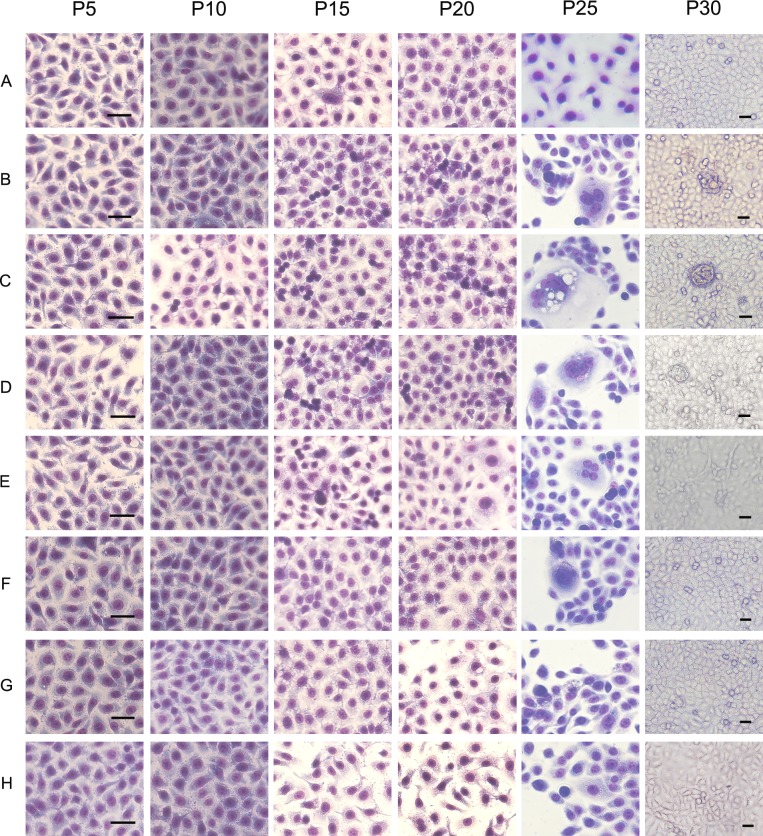
Cell morphology of human bronchial epithelial cells treated with BaP in the bionic lung chip culture A, control group (DMSO); B, BaP-treated group; C, BaP + coculture with macrophages group; D, BaP + coculture with macrophages + anti-IL-6 antibody treatment group; E, BaP + coculture with macrophages + anti-TNF-α antibody treatment group; F, BaP + coculture with macrophages + NF-κB inhibitor transfection group; G, BaP + coculture with macrophages + STAT3 inhibitor transfection group; H, BaP + coculture with macrophages + cyclinD1 inhibitor transfection group. Scale bar, 20 μm.

Next, we utilized the bionic airway chip to mimic the *in vivo* conditions to assess the role of BaP, macrophages, and different genes in the malignant transformation of human bronchial epithelial cells. After the cells were exposed to BaP, the morphology of the 16HBE cells showed significant changes compared to the untreated cells (Fig. 3); i.e., some cells showed condensed nuclei and abnormal nuclear-cytoplasmic ratios accompanied by atypical mitoses (Fig. 3B), compared to the untreated cells (Fig. 3A). The appearance of abnormal cells started to occur in passage 15 in the BaP group (Fig. 3B), whereas it occurred in passage 10 in the coculture of BaP and macrophages group (Fig. 3C). However, blockage of IL-6 or TNF-α signaling or inhibition of NF-κB, STAT3, or cyclinD1 expression could prolong the time when abnormal cells began to appear (Fig. 3D–H). Furthermore, the cells in the BaP group and the coculture of BaP and macrophages group showed multiple layers of cell growth, an indication of lost cell contact inhibition in cells at passage 30; however, this cell growth was not observed in the coculture of BaP and macrophages groups treated with inhibitors.

**Figure 4:**
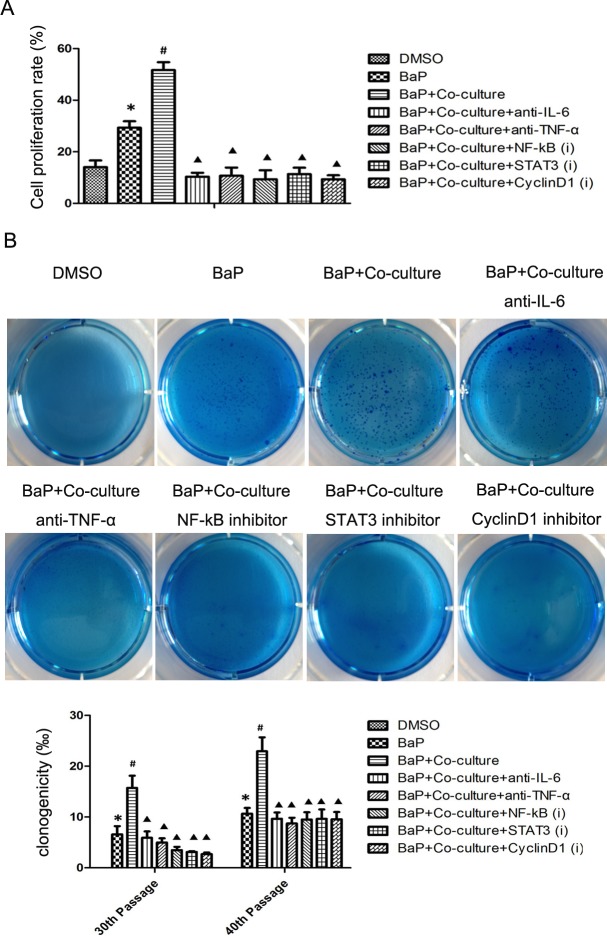
Cell proliferation and colony formation data on human bronchial epithelial cells after BaP treatment A, Cell proliferation assay. 16HBE cells at passage 30 were subjected to the cell proliferation assay after exposure to BaP. B, Colony formation assay. 16HBE cells at passages 30 and 40 were subjected to the colony formation assay after exposure to BaP. *p < 0.05 compared to the DMSO group, ^#^*p* < 0.05 compared to the BaP group, ^▲^*p* < 0.05 compared to the BaP + coculture with macrophages group.

Tumorigenicity is characterized by growth promotion. As shown in Fig. 4A, BaP treatment could stimulate bronchial epithelial cell proliferation, while macrophages also enhanced this effect. As expected, blockage of cytokine IL-6 or TNF-α signaling or inhibition of NF-κB, STAT3, or cyclinD1 expression inhibited cell proliferation.

A cell colony formation assay using cells at passages 30 and 40 in each group showed that the colony formation efficiency in the BaP plus coculture group was significantly higher than that in the BaP group and the BaP plus coculture and inhibitor groups (*p* < 0.05; Fig. 4B), indicating that macrophages could promote the malignant transformation of 16HBE cells, whereas blockage of cytokine IL-6 or TNF-α signaling or inhibition of NF-κB, STAT3, or cyclinD1 expression could abrogate the effects of BaP or macrophages on the promotion of malignant transformation of 16HBE cells.

**Figure 5:**
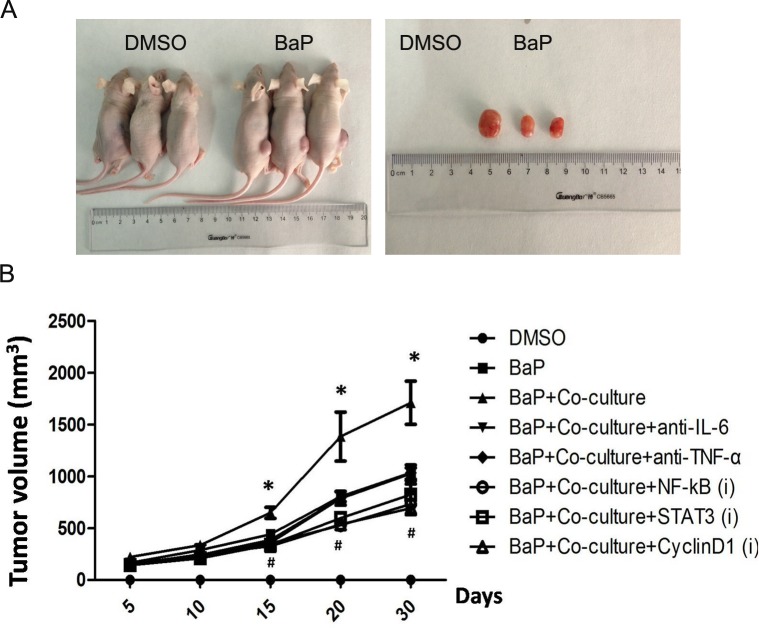
Tumorigenicity of the transformed 16HBE cells in nude mice A, Representative images of tumor formation on the 30^th^ day after cell transplantation. B, Growth curves of tumors in nude mice. *p < 0.05 compared to the BaP group, ^#^*p* < 0.05 compared to the BaP + coculture with macrophages group.

In addition, we also assessed their *in vivo* effects using a nude mouse model of tumor formation. Thirty days after cell transplantation into nude mice, there was no tumor formation in the DMSO group, whereas tumors did form in the BaP-treated group (Fig. 5A). The tumor growth curve showed that the average tumor volume in the BaP plus coculture group was larger than that of the same passage of 16HBE cells alone exposed to BaP at different observation time points (*p* < 0.05). However, blockage of cytokine IL-6 or TNF-α signaling or inhibition of NF-κB, STAT3, or cyclinD1 expression reduced the average tumor volume (Fig. 5B; *p* < 0.05).

### Macrophages enhanced NF-κB and STAT3 signaling in the malignant transformation of bronchial epithelial cells *in vitro* and *in vivo*

**Figure 6:**
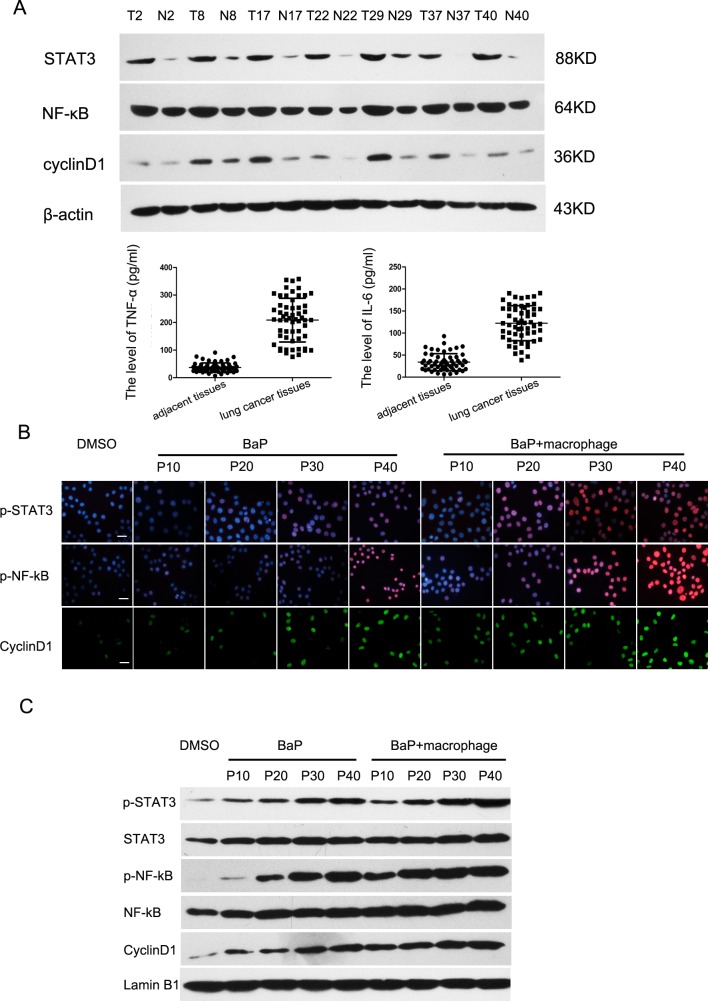
Activation of the NF-κB and STAT3 signaling pathways in lung cancer A, Western blot. Expression levels of TNF-α, IL-6, NF-κB, STAT3, and cyclinD1 proteins in lung cancer tissues. B, Immunofluorescence assay. Proteins of the NF-κB and STAT3 signaling pathways were detected during the malignant transformation of 16HBE cells by an immunofluorescence assay. Scale bar, 10 μm. C, Western blot. Proteins of the NF-κB and STAT3 signaling pathways were assayed during the malignant transformation of 16HBE cells by western blot.

To demonstrate the critical role of the TNF-α/NF-κB/cyclinD1 and IL-6/STAT3/cyclinD1 pathways in the malignant transformation of 16HBE cells, we first detected the expression levels of TNF-α, IL-6, NF-κB, STAT3, and cyclinD1 in lung cancer tissues. As shown in Fig. 6A, NF-κB, STAT3, and cyclinD1 were expressed in almost all of the cancerous and adjacent lung tissues. However, the expression of NF-κB, STAT3, and cyclinD1 in the lung cancer tissues was significantly higher than that in the adjacent tissues. Furthermore, we dynamically detected the protein levels of the above signaling molecules in cells cultured in the bionic airway chip. NF-κB, STAT3, and cyclinD1 proteins were activated in the bronchial epithelial cells from higher passages after exposure to BaP. Furthermore, macrophages were able to activate NF-κB, STAT3, and cyclinD1 proteins (Fig. 6B). In addition, the expression of NF-κB, STAT3, and cyclinD1 proteins was induced in different passages of cells after exposure to BaP (Fig. 6C).

**Figure 7:**
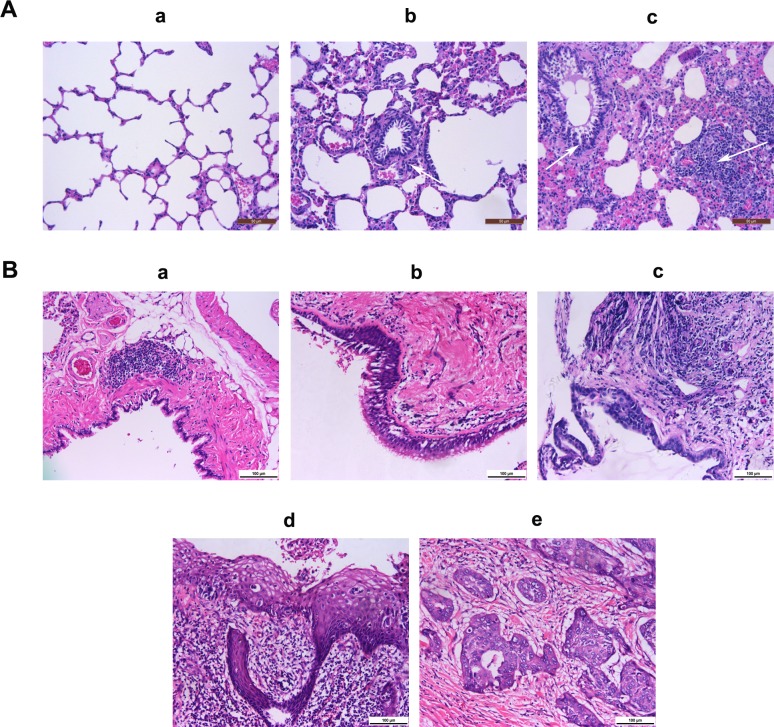
Representative images of airway inflammation and tumorigenesis in rats A, The airway inflammation in a model stimulated by cigarette smoke. (a) A lung tissue section from a normal rat; (b & c) a lung tissue section from a rat exposed to cigarette smoke. Scale bar, 50 μm. B, The airway tumorigenesis model induced by BaP. (a) Normal bronchial epithelium; (b) squamous metaplasia; (c) atypical hyperplasia; (d) carcinoma *in situ*; (e) invasive carcinoma. Scale bar, 100 μm.

Indeed, we found that macrophages were able to promote the malignant transformation of bronchial epithelial cells *in vivo*. First of all, we fabricated a chronic lung inflammation model by using cigarette smoke. As shown in Fig. 7A, the alveolar wall became thickened and congested, and the small airway was narrow and infiltrated with inflammatory cells (b and c, white arrows), compared to the normal rat lung (a). To confirm the role of macrophages and the related signaling pathways in rat lung tumorigenesis, we depleted macrophages and knocked down the key factors in these signaling pathways. As shown in Fig. 7B, a chemical carcinogen-induced rat lung cancer model was successfully simulated. The rat lung cancer model presented the following pathological characteristics: normal bronchial epithelium (a), squamous metaplasia (b), atypical hyperplasia (c), carcinoma *in situ* (d), and invasive carcinoma (e). Finally, as shown in Table 2, the tumorigenic rate in the MCA+DEN group was 37.5% vs. no lung tumors found in the blank control and inflammation groups. However, when macrophages were depleted, the tumorigenic rate was significantly decreased (25.0% vs. 85.7%). Furthermore, when the NF-κB, STAT3, and cyclinD1 expression levels were knocked down, the tumorigenic rates also showed different degrees of reduction.

**Table 2:** Rate and number of tumors in Wistar rats

Group	Total number	Tumor number	Tumorigenic rate(%)
Control		0	0
	10		
Inflammation	8	0	0
MCA+DEN	8	3	37.5
Inflammation+MCA+DEN	7	6	85.7
Inflammation+MCA+DEN+Mo (i)	8	2	25.0
Inflammation+MCA+DEN+NF-κB (i)	9	2	22.2
Inflammation+MCA+DEN+STAT3		3	30.0
(i)	10		
Inflammation+MCA+DEN+cyclinD1		4	40.0
(i)	10		

Note: MCA, 3-methylcholanthrene; DEN, diethylnitrosamine; (i), inhibitor.

## DISCUSSION

In the current study, we first detected the level of macrophages in the cancerous and adjacent lung tissues and then fabricated a bionic lung chip that allowed the coculture of normal bronchial cells and macrophages treated with or without BaP or different inhibitors or siRNAs. After that, we confirmed our current *in vitro* data in animal models. Our current study led to a number of novel discoveries. First, macrophages were critical to promote carcinogen-induced lung tumorigenesis. Second, the TNF-α/NF-κB/cyclinD1 and IL-6/STAT3/cyclinD1 signaling pathways were primarily responsible for the regulation of macrophage-promoted lung tumorigenesis. Third, the bionic lung chip was an excellent platform to investigate the cell–cell interactions that mimic the *in vivo* situation. Our current data extended previous findings and provided a novel technology for the *in vitro* study of cell–cell interactions and gene regulations *in vivo*.

Indeed, many previous studies have demonstrated that macrophages are able to support the growth of breast, lung, and ovarian cancer in animal models. Our current study provided evidence for the role of macrophages in promoting lung tumor development, although previously, macrophages have been commonly considered as a killer of cancer cells due to their cytotoxic and phagocytic activities. In our current study, we revealed that macrophages have a strong protumorigenic function *in vitro* and *in vivo*. In a previous study, Rinat *et al.* reported that macrophages could promote lung tumorigenesis in urethane-induced mice [22]. In addition, our current study verified this notion based on both a bionic airway model and animal experiments. To the best of our knowledge, this is the first study regarding malignant transformation of bronchial epithelial cells using a microfluidic chip culture. We also found that macrophages were able to speed up the course of malignant transformation of bronchial epithelial cells.

Furthermore, our current study also showed that the downregulation of NF-κB, STAT3, or cyclinD1 expression or blockage of IL-6 or TNF-α activity could abrogate the malignant transformation of bronchial epithelial cells *in vitro* or decrease lung tumorigenesis *in vivo*. In the bionic airway model *in vitro*, the expression of IL-6, TNF-α, NF-κB, STAT3, or cyclinD1 was upregulated at the onset of carcinogen exposure. Cell morphology, proliferation, anchor-independent growth, and tumor formation in nude mice were dynamically obvious. These results indicated that IL-6, TNF-α, NF-κB, STAT3, and cyclinD1 were the key factors during malignant transformation of bronchial epithelial cells. In the rat lung cancer model, the expression of NF-κB, STAT3, or cyclinD1 was knocked down before carcinogen exposure and the tumorigenic rate was decreased by varying degrees accordingly. These findings suggested that the TNF-α/NF-κB/cyclinD1 and IL-6/STAT3/cyclinD1 signaling pathways are two potential pathways in the course of tumorigenesis mediated by macrophages. A previous study has shown that M1 macrophage polarization is associated with a good prognosis for lung cancer patients [23]. Despite the fact that polarized M1 macrophages are considered to have antitumor activity, persistent activation of these cells may lead to cell damage, thereby causing malignant transformation [24]. Pro-inflammatory cytokines produced by M1 macrophages, such as IL-6 and TNF-α, have been reported to protect initiated epithelial cells from apoptosis and promote the proliferation of these cells [25]. Our current study showed that the IL-6 and TNF-α protein levels were significantly higher in the 16HBE cell and macrophage coculture than those in 16HBE cells only in the bionic airway model. In addition, NF-κB, STAT3, and cyclinD1 proteins were gradually activated in bronchial epithelial cells at higher passage numbers. Therefore, we speculated that TNF-α and IL-6 secreted from macrophages could activate NF-κB, STAT3, and their downstream proteins, such as cyclinD1, thereby stimulating cell proliferation and tumorigenesis. Indeed, tumor cells acquire a number of characteristic alterations during the course of malignant transformation, such as a capacity for uncontrolled proliferation, tumor cell invasion into the surrounding tissues, and metastasis [6]. These properties are initiated, at least in part, by alterations of cell signaling pathways, including MAPK, PI3K, and NF-κB, which control cell proliferation, motility, and survival. For example, the NF-κB pathway plays a vital role in the malignant transformation of cells in chronic colitis and hepatitis [3, 26]. However, there are many key signaling molecules and pathways involved in the tumorigenesis of lung cancer with chronic airway inflammation related to macrophages. When chronic inflammation occurs, the airways are infiltrated with macrophages and pro-inflammatory cytokines, such as IL-1β, IL-6, and TNF-α, which in turn activate the MAPK, PI3K, and NF-κB pathways [27]. Indeed, previous studies have shown that certain immunosuppressants such as rapamycin are useful in cancer prevention and treatment, although others argue that the use of immunosuppressants may be associated with an increased risk of cancer [28]. Moreover, rapalogs such as rapamycin have been used as a novel strategy for cancer prevention because of their anti-aging or anticancer activities [29]. Thus, the use of these agents to manipulate these gene pathways could effectively prevent lung carcinogenesis, but further study is warranted.

The bionic airway model used in this study was a unique and ideal platform to investigate the cell–cell interactions involved in the malignant transformation of bronchial epithelial cells because this model vividly simulates the real structure and microenvironment of the airways in humans. Currently, the challenge of the organ-on-chip model is to recapitulate the healthy and diseased physiology of multiple tissues or organs to mimic the *in vivo* conditions in order to supplement animal studies for preclinical drug development [30, 31]. Developing such platforms could allow us to robustly mimic reliable tissue or organ-level structural and functional outputs while maintaining the simplicity required for high-throughput systems. In this study, we designed, built, and tested an *in vitro* model of healthy and carcinogenic human airway structures. Using this human bionic airway chip culture system, we demonstrated that we could recapitulate the whole course of lung tumorigenesis. Compared to other *in vitro* models, this novel platform has the following advantages: its minute structure, high-oxygen permeable PDMS materials, and a continuous flow of air and medium that can mimic an *in vivo* microenvironment and meet the requirements for human bronchial epithelial cell culture. Therefore, compared to conventional platforms, the research results from the microfluidic chip may be more meaningful. Furthermore, this microfluidic chip can be suitable for high-throughput screening for potential signaling pathways, therapeutic targets, and time-lapse imaging. More importantly, the microfluidic chip allowed both morphological analysis and fluorescence marker detection to fulfill the dynamic observation of cultured cells and their signaling during the long-term exposure of a carcinogen. Thus, this designed microfluidic chip is a unique platform to study cell–cell interactions in the malignant transformation of bronchial epithelial cells.

However, our current study does have some limitations; for example, we only studied the interactions of macrophages with bronchial cells in this bionic airway chip culture and future research should incorporate additional cell types (such as fibroblasts or endothelial cells) into this system to study the cell–cell interactions. Moreover, the underlying molecular mechanism by which macrophages with or without BaP treatment interact with bronchial cells is preliminary, and further study is needed to clarify the role of the TNF-α/NF-κB/cyclinD1 and IL-6/STAT3/cyclinD1 signaling pathways on the regulation of malignant transformation of bronchial epithelial cells.

## MATERIALS AND METHODS

### Tissue specimens

This study was approved by the Institutional Review Board and Human Ethics Committee of The Second Affiliated Hospital of Dalian Medical University, and a written informed consent form was obtained from individual patients. Briefly, a total of 57 lung cancer patients with chronic obstructive pulmonary disease were recruited from The Second Hospital, Dalian Medical University (Dalian, China), between January 2013 and August 2013. The demographic and clinical data are shown in Supplementary Table 1. The lung cancer tissues and adjacent tissues after surgical removal were isolated from the primary tumor and stored in liquid nitrogen.

### Immunohistochemistry

The distribution of macrophages in the lung cancer tissues and adjacent tissues was assessed by immunostaining of CD68 protein, an established marker of macrophages, with an antibody against CD68 and the SP-9000 Histostain^TM^-Plus Kit (ZYMED, MA, USA), as described previously [32]. The immunostained tissue sections were reviewed and scored independently under a microscope by two investigators. Specifically, staining of CD68 expression was visualized as a brown color in the cytoplasm and estimated by averaging the number of positive-stained cells under 10 high-power vision fields.

### Protein extraction and western blot

Protein lysates from cancerous and normal lung tissues as well as 16HBE cells were separated by sodium dodecyl sulfate–polyacrylamide gel electrophoresis (SDS-PAGE) and transferred onto nitrocellulose membranes (BioTrace^TM^, Pall Corporation, San Diego, CA, USA). After blocking with 5% skim milk in phosphate-buffered saline (PBS), the membranes were incubated with a primary antibody against the selected key proteins, including NF-κB p65 or phospho-NF-κB p65 (Ser536) (1:1000; Abcam, Cambridge, UK), STAT3 or phospho-STAT3 (Ser727) (1:1000; Abcam), cyclinD1 (1:1000; Abcam), β-actin (1:5000; Proteintech Group Inc., Chicago, IL, USA) or Lamin B1 (1:5000; Abcam), and the bound antibodies were detected by using a Super Signal West Pico Kit (Thermo Fisher Scientific Inc., USA), followed by quantitative densitometric analysis using Eagle Eye II software.

### Construction and fabrication of the bionic airway chip

To mimic the cell compartmentalization of the airway in humans (Fig. 1A), we fabricated a working biomimetic airway chip. The main body of the chip consisted of three parts: two polydimethylsiloxane (PDMS) layers (the top and bottom layers) and one porous membrane layer (the middle layer) (Fig. 1B). The PDMS microfluidic chip was fabricated using a Sylgard 184 kit (Dow Corning, Midland, TX, USA) by a standard soft lithography method. Briefly, two PDMS layers were fabricated by replica molding against masters, and a 5-μm pore polycarbonate membrane (Nuclepore, Whatman, Buckinghamshire, UK) was bonded using wet aminosilanization of the membrane and contact binding with oxygen plasma-treated PDMS (150 mTorr, 50 W, 25 s). The microfabricated device was then continuously supplied with air and cell growth medium to mimic the real microenvironment of the bronchi in human lungs. The human bronchial epithelial cell line 16HBE (obtained from the Chinese Academy of Medical Sciences, Beijing, China) and macrophages were cultured on the top and bottom of the porous membrane, respectively. The ratio of 16HBE cells and macrophages was 10:1 (16HBE cells/macrophages). The upper channel pumped sterile air, and the bottom channel simultaneously pumped cell culture medium using a syringe pump.

### Establishment of the BaP-induced tumorigenic transformation model

According to methods reported previously [33], 16HBE cells were continuously exposed to 10 μM BaP (Sigma-Aldrich, St. Louis, MO, USA, diluted in dimethyl sulfoxide, DMSO) in serum-free medium with a mouse liver S9 mixture (drug-metabolizing enzymes, carcinogen activators) for 4 h, respectively, every 7 days for 5 weeks. 16HBE cells exposed to DMSO were used as a vector control. 16HBE cells were cultured under these conditions for 40 passages. During this period of time, 16HBE cells were collected from the channels of the bionic airway chip and assayed for cell proliferation, migration, invasion, colony formation, and tumorigenicity in nude mice.

To determine the role of macrophages in the malignant transformation of bronchial epithelial cells, macrophages were added into the BaP-induced tumorigenic transformation model. Briefly, the BaP-induced 16HBE cells were cultured with or without macrophages at a ratio of 10:1 (16HBE cells/macrophages) in each passage of 16HBE cells. The time of tumor appearance and the severity of related tumorigenic indicators such as cell proliferation and anchor-independent growth were compared.

To demonstrate the role of the NF-κB and STAT3 signaling pathways in the malignant transformation of bronchial epithelial cells, inhibition of the key signaling molecules was performed in this model. We introduced IL-6 neutralizing antibody (1:400, Abcam, ab6672), TNF-α neutralizing antibody (1:200, Abcam, ab9635), NF-κB inhibitor BAY11-7082 (2 μM, InvivoGen), STAT3 inhibitor S31-201 (100 μM, Selleck, Houston, TX, USA), or cyclinD1 inhibitor CDK4 inhibitor (50 nM, Santa Cruz Biotechnology, Santa Cruz, CA, USA) into the microfluidic channels incubated with the cells for 24 h in each passage after the last BaP exposure.

### Cell morphology observation

16HBE cells were collected from the channels every five passages, starting from passage 5 to passage 30, then fixed with ice cold methanol, stained with Giemsa stain, and observed under an inverted microscope.

### Cell proliferation assay

16HBE cells at passages 20 and 30 were collected, and cell proliferation was determined using a Cell-Light EdU Apollo 567 *in vitro* Kit (Ribobio, Guangzhou Ribobio Company, China), according to the manufacturer's instructions. Briefly, cells were labeled with EdU, fixed with paraformaldehyde, stained for Apollo and DNA, and reviewed under a fluorescence microscope (Nikon Eclipse Ti, Japan).

### Cell anchor-independent growth assay

A soft agar assay was performed to assess the ability of 16HBE cells at passages 30 and 40 to grow on soft agar. The cells at a density of 2000/well were cultured for 2 weeks in 0.3% low-melting point agarose (Sigma-Aldrich) in complete medium that was overlaid on 0.6% agarose in six-well plates. Individual cell colonies with more than 50 cells were counted under a microscope.

### Tumor formation assay in nude mice

Nude mice were utilized to assess tumor formation *in vivo*. Specifically, BALB/c-nu nude mice (4 weeks old) were purchased from the Laboratory Animal Centre, Dalian Medical University, and the animal study was approved by the Life Sciences Institutional Review Board of Dalian Medical University. The transformed 16HBE cells (5 × 10^6^/ml) in 200 μL of PBS were injected into the back of nude mice (n = 3 per group). The tumor size was calculated according to the formula V = L × W^2^ / 2 every 5 days after cell inoculation. The tumor was removed and weighed on the 30th day.

### Detection of NF-κB and STAT3 expression in the BaP-induced malignant transformation of 16HBE cells

The expression and localization of NF-κB and STAT3 signaling pathway proteins were assessed by either immunofluorescence or western blotting. For immunofluorescence staining, 16HBE cells, BaP-induced 16HBE cells, and BaP-induced 16HBE cells cultured with macrophages at passages 10, 20, 30, and 40 were collected from the channels of the bionic airway chip, fixed, permeabilized, stained with 1:200 diluted phospho-NF-κB p65 (Ser536) antibody (Abcam), phospho-STAT3 (Ser727) antibody (Abcam), and cyclinD1 antibody (Abcam), and visualized using AlexaFluor488 or AlexaFluor594-labeled secondary antibodies, followed by review and scoring under a laser scanning confocal microscope (Leica TCS SP5, Mannheim, Germany).

### Macrophage promotion of carcinogen-induced malignant transformation in a rat lung cancer model

Eighty male Wistar rats (6 weeks old, 180 ± 20 g) were purchased from the Laboratory Animal Center of Dalian Medical University. The experiments were approved by the Laboratory Animals Committee of Dalian Medical University. Sixty rats were instilled with the carcinogens 3-methylcholanthrene (MCA, 10 mg) and diethylnitrosamine (DEN, 10 μl) (Sigma–Aldrich) in an iodized oil mixture into the left lung using a method established previously [34]; 10 rats were left untreated as a blank control. At the end of the experiment, the rats were euthanized under anesthesia and the whole lungs were immediately excised. The left lungs were fixed with 4% formaldehyde in PBS for 24 h, embedded in paraffin, and subjected to hematoxylin-eosin staining.

To investigate the role of macrophages in rat lung carcinogenesis, 50 carcinogen-instilled rats were exposed to cigarette smoke for 2 h each day for 3 months, and the remaining 10 carcinogen-instilled rats were untreated as a positive control. In addition, 10 rats were only exposed to cigarette smoke as a smoking control. Furthermore, macrophages were depleted by instilling clodronate (Sigma-Aldrich)-containing liposomes as described previously [22].

Moreover, to determine the role of the NF-κB and STAT3 pathways in rat lung carcinogenesis, we knocked down the expression of NF-κB, STAT3, and cyclinD1 by injection of NF-κB, STAT3, and cyclinD1 siRNA (Santa Cruz Biotechnology) loaded with the Entranster^TM^-in Transfection Reagent (Engreen Biosystem Co., Ltd., Beijing, China) through the caudal vein every week for 3 months.

### Statistical analyses

All experiments were performed at least three times. Data were expressed as the means ± standard deviation. The difference among groups was analyzed by analysis of variance using SPSS13.0 for Windows software (SPSS, Chicago, IL, USA). A *p* value less than 0.05 was considered statistically significant.
